# Miscellany

**Published:** 1897-11

**Authors:** 


### Miscellany.

### ADULTERATION OF FOOD AND DRUGS.

The Secretary of Agriculture, in pursuing an inquiry into the subject, has issued the following circular :

United States Department of Agriculture,
Division of Chemistry,
Washington, D C., September 17, 1897.

*Dear Sir*—Under authority of Congress, the department of agriculture is investigating the extent and character of food and drug adulterations and is desirous of securing all the information possible on the subject. Having been appointed special agent to inquire into and report upon this matter, the undersigned writes to request that you kindly furnish the Department under the inclosed franks all the information you have in regard to adulterations, together with any suggestions as to the best remedy for the evil.

1 Do you know of any new adulterant ? If yes, state what and how used.

2. Would a national food and drug law assist in preventing adulteration ?

3. Would uniform food, drug and pharmaceutical laws tend to promote efficiency and purity ?

4. Please suggest what would best promote the interests of consumers and legitimate manufacturers and dealers ?

5. What is your opinion as to the extent of damage done legitimate business by imitation of brands, packages, and the like ?

6. To what extent do sophistication, misbranding and injurious adulteration exist ?

7. Have state laws aided in preventing adulteration ? To what extent ?

8. Would a national law assist state officials in properly executing the local laws ?

9. Have adulteration, sophistication and misbranding increased or decreased ?

Prompt replies to the above, together with any other information or suggestions, will be highly appreciated.

Yours respectfully,

A. J. WEDDERBURN, *Special Agent.*

The circular bears the approval of the secretary, together with the request that answers be written on the reverse side of the circular and returned to the special agent.

---

### SHAKESPEARE REVISED.

By D. S. MADDOX, M. D.

(From the *Quarterly Medical Journal*, Sheffield, England.)

"Constipation, thy name is woman."—*Hamlet.*

"Nature hath made strange cures in her time."—*Merchant of Venice.*

"Take each man's fee, but reserve thy diagnosis."—*Hamlet.*

"How poor are they that have not patients!"—*Othello.*

"He jests at physic that never felt a pain."—*Romeo and Juliet.*

"Why should the poor be pauperised."—*Hamlet.*

"Consultation oft loses both fee and patient."—*Hamlet.*

"It is a wise doctor that knows his own patient."—*Merchant of Venice.*

"Give every man thy prescription, but few thy medicine."—*Hamlet.*

"Doctors wax poor when patients prove unkind."—*Hamlet.*

"All the world's a clinic,
And all the men and women merely patients."—*As You Like It.*

"Thou art not so unkind
As a patient's ingratitude."—*As You Like It.*

"He that is not guilty of taking his own physic shortens not his own life."—*Hamlet.*

"Oh! me, what doctor that roars so loud,
And thunders in the index?"—*Hamlet.*

"I do much like her. She doth think she has
Strange lingering diseases."—*Cymbeline.*

"A double fee is a double grace,
Occasion smiles upon a second call."—*Hamlet.*

"What misadventure is so early up,
That calls the doctor from his morning's rest?"—*Romeo and Juliet.*

"If it were opened when 'tis opened, then 'twere well it were opened quickly."—*Macbeth.*

"Make all our trumpets talk, give them more wind,
Those clamorous harbingers of check and sound."—*Macbeth.*

"And all our theories have lighted fools
The way to dusty death."—*Macbeth.*

"What damned error, but some sober brow
Will approve it, and it with a case."
—*Merchant of Venice.*

"The remedies thou hast and their adoption tried,
Apply them to thy case with zeal,
But do not kill thy patients with experiment
Of each new-hatched unfledged medicine."—*Hamlet.*

—*The Cincinnati Lancet-Clinic.*

